# Thin Layer Sonoelectrochemistry: The Solvents

**DOI:** 10.1021/acs.jpcc.4c08175

**Published:** 2025-02-28

**Authors:** Nadeesha P. W. Rathuwadu, Daniel L. Parr, Johna Leddy

**Affiliations:** Department of Chemistry, University of Iowa, Iowa City, Iowa 52240 United States

## Abstract

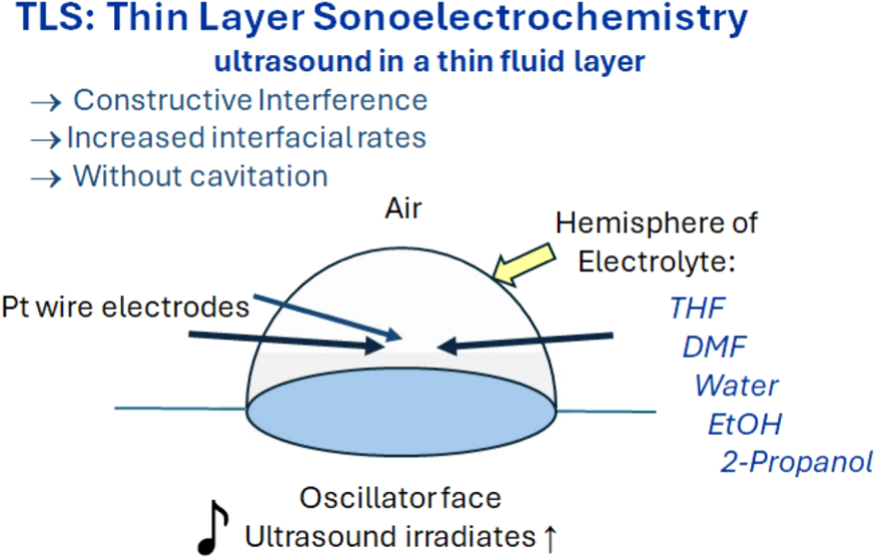

In thin layer sonoelectrochemistry (TLS), ultrasound induces constructive interference in a thin fluid layer to increase interfacial rates. In TLS experiments, slow interfacial rates are increased during and after sonication. No cavitation or heating is observed in the fluid. A previously developed model quantifies how solvent properties impact TLS rates. Voltammetry for Fe^3+^ and benzoquinone in tetrahydrofuran, dimethylformamide, water, ethanol, and 2-propanol is undertaken with and without sonication. Rate enhancements vary with solvent properties, as quantitatively predicted by the model. The data vet the TLS model for nonaqueous solvents.

## Introduction

In thin layer sonoelectrochemistry (TLS), ultrasound is applied to a thin layer of fluid that contains an electrode parallel to the face of an ultrasonic transducer.^1−3^ In a properly configured TLS cell,^4^ constructive interference^5,6^ builds sound pressure energy at the electrode | electrolyte interface to increase electron transfer rates^2^ and remove oxide layers.^3^ Classical sonochemistry (CS) is undertaken in bulk fluids,^7−22^ where cavitation and fluid heating are observed. In TLS, no cavitation and no temperature changes (≲1 °C) are found.^2,3^ From voltammetry, no impacts on transport are observed.^2,3^ In TLS, sound pressure energy is focused at the electrode | electrolyte interface, so energy is not dissipated as cavitation and heat in the electrolyte.

From the model for TLS, the thickness of the thin fluid layer  and the wavelength of the ultrasonic transducer λ are matched to establish constructive interference at the electrode | electrolyte interface.^4,5^ The model characterizes how the physical properties of the fluid and electrode material, as well as the oscillator frequency, amplify ultrasonic sound pressure at the interface. The model provides a solvent-specific analytical expression to establish the constructive interference that focuses energy at the interface to impact slow electrode reactions. Prior work used water as the solvent.^1−3^

Here, the model is further vetted against TLS in nonaqueous, electrochemical solvents.^23^ The nonaqueous electrochemical solvents are tetrahydrofuran (THF), dimethylformamide (DMF), ethanol (EtOH), and 2-propanol. TLS in water and the four nonaqueous solvents is evaluated by cyclic voltammetry (CV) with and without sonication for two redox probes, ferric ion (Fe^3+^) and benzoquinone (BQ). Voltammograms are recorded before sonication (PreSono), during sonication (Sono), and after sonication (PostSono). Slow interfacial electron transfer rates are impacted. Currents and rates increase with sonication. As previously, CVs of tris(bipyridine)ruthenium (Ru(bpy)_3_^2+^) in water are unaltered on sonication.^2,3^ For Fe^3+^ in nonaqueous solvents, currents and rates are increased during and after sonication. Although mechanisms vary for BQ, currents increase with sonication. Impacts of sonication persist for at least 10 min after sonication ceases. For Fe^3+^ and BQ in nonaqueous solvents, sonicated current is amplified relative to current prior to sonication. Amplification across solvents correlates linearly with the solvent properties that establish transmissibility (amplification), as anticipated by the model. The linear correlation vets the TLS model for nonaqueous solvents.

## Background

In classical sonochemistry, high-energy sonic transducers are placed in bulk solution where cavitation and heating are observed.^7,9,18−22^ Where cavitation arises, substantial temperature and pressure excursions are observed at the narrow interface where cavitation bubbles collapse.^18−22^ Cavitation impacts various chemical processes such as electrodeposition and the synthesis of nanomaterials. However, cavitation and heating represent large thermal losses, as input energy is dissipated randomly throughout the electrolyte. Cavitation bubbles disrupt mass transport chaotically.

**Figure 1:**
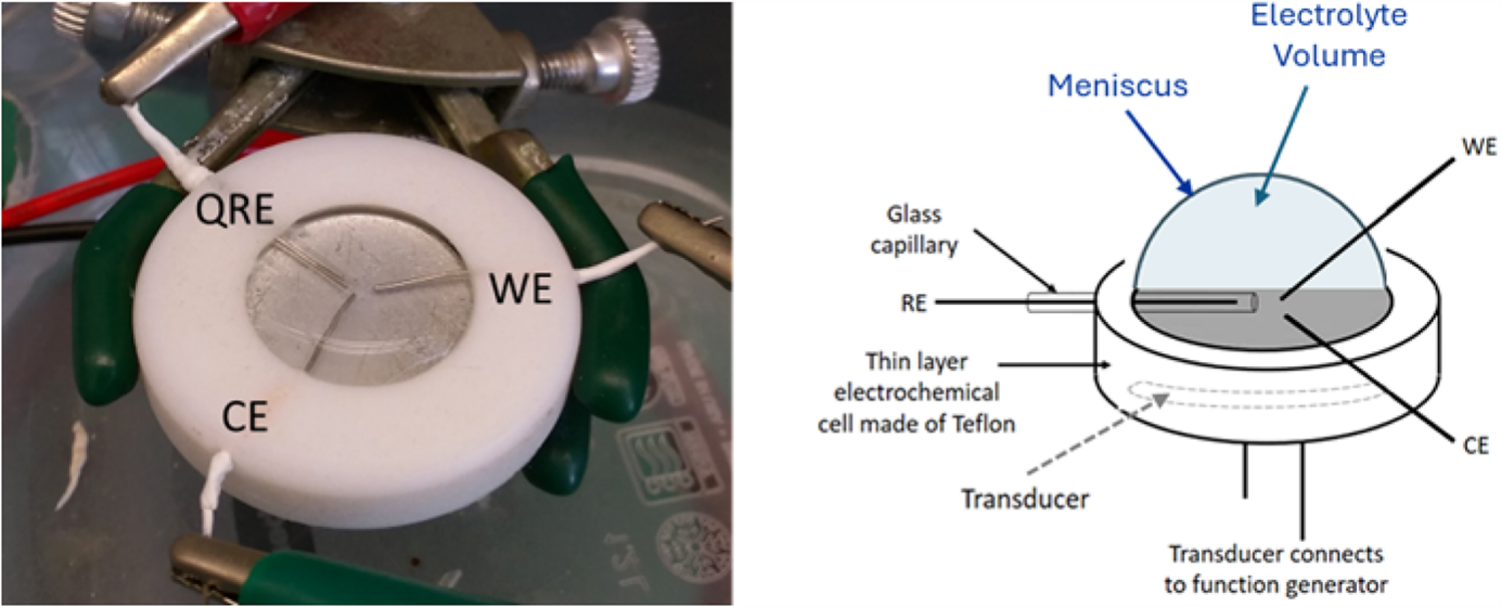
In the photograph of the thin layer cell, the 41 kHz QCO (transducer) forms the bottom of the well. The quasireference electrode (QRE) housed in a glass capillary, working electrode (WE), and counter electrode (CE) are Pt wires. In the diagram, the transducer is embedded from the bottom of the Teflon hollow cylinder. Leads to the function generator that drives the QCO are shown. A hemispherical electrolyte meniscus at the air electrolyte interface is shown above the face of the transducer.

Initial TLS experiments were made with a hemisphere of aqueous electrolyte above the face of a 41 kHz quartz crystal oscillator (QCO).^1−3^ Three platinum wires served as the electrodes, as shown in Figure 1. Unexpectedly, no cavitation was observed and the electrolyte did not change temperature over many minutes of sonication. From voltammetry, the electron transfer rate for ferric ion increased on sonication, but the voltammetry for Ru(bpy)_3_^2+^ was unchanged (Figure SI.2). An outer sphere probe, electron transfer for Ru(bpy)_3_^2|3+^ is fast. Fe^2|3+^ is marginally an inner sphere probe with slower electron transfer rates because the number of water of hydration differs for Fe^3+^ and Fe^2+^. TLS increases the rates for slow electron transfers but does not alter the rates for fast interfacial processes. Of note, the impacts of sonication on electron transfer rates persist for at least 20 min after the sonication ceases.^2,3^ Sonication under thin layer conditions does not induce convection and does not impact mass transport. In TLS experiments,^3^ sonication of platinum wire electrodes removes platinum oxide after several minutes of sonication, and oxygen reduction rates at platinum in acidic electrolyte increase on sonication. Advantages of TLS include the QCOs that require little input energy and can be 97% transduction efficient. Thin layer cell construction does require attention to cell geometry.^4^

**Figure 2:**
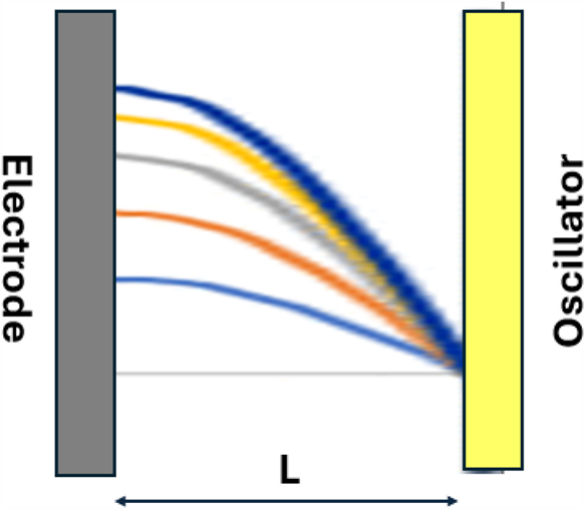
Constructive interference is established in thin fluid layer when the parallel plates of electrode and oscillators are separated by a distance *L* that is matched to the wavelength of the oscillator.^5^ Constructive interference focuses energy at the electrode–electrolyte interface to increase rates of slow interfacial reactions. Sinusoidal sound pressure waves of different oscillator intensities are shown where sound pressure is reflected from the electrode surface , and *L* = λ/4. Properties of the electrode material and the solvent impact transmissibility (amplification) at the electrode | solution interface.

A model identifies constructive interference as effective in a thin fluid layer.^5,6^ The one dimensional model for sonication is for a thin fluid layer of thickness *L* between parallel faces of the electrode (*x* = 0) and sonicator (*x* = *L*), as shown in Figure 2. Sound pressure *u*(*x, t*) is established in the fluid by the energy input from the oscillator. Where sound pressure is reflected from the electrode surface, energy is selectively deposited at the electrode | electrolyte interface. Conditions for constructive interference are established where the sound pressure is underdamped and no cavitation is generated. In water at platinum where constructive interference is established with an ultrasonic oscillator, the energy delivery rate is estimated at ∼1 kJ (mol s)^−1^. To achieve underdamping, the oscillator wavelength and the cell thickness are comparable and must be appropriately matched.^5^ Geometric constraints on thin layer cell design derive from the model.^4^

Thin layer sonochemistry applies to all systems where the oscillator wavelength and fluid layer thickness are well matched. For ultrasound, the layer thickness is on the order of a centimeter. Thin layer sonochemistry depends on solvent properties of density ρ and viscosity η, and speed of sound *c* in the solvent, sonicator frequency *f* or radial frequency ω, and reflectivity *R*_solvent | electrode_ of the solvent | electrode interface.^5^ Solvent properties set the transmissibility *T* that characterizes sound pressure amplification.

### Characteristic Equations

In the one dimensional model for constructive interference, the thickness of the thin fluid layer is *L*. The sonicator is positioned at *x* = *L* and generates a sinusoidal sound pressure *u*(*L, t*) wave that is reflected off the electrode at *x* = 0. See Figure 2. Constructive interference is established at the electrode | electrolyte interface when sound pressure is efficiently reflected from the electrode surface (*x* = 0) to compound sound pressure energy at the interface. Maximum reflectivity occurs where the sound pressure gradient is zero at *x* = 0, ∂*u*(*x*, *t*)/∂*x*|_*x*=0_ = 0.

The fundamental equations for constructive interference of sound pressure in a thin fluid layer are presented.^5^ Experimental conditions necessary to establish constructive interference in TLS are provided in ref (4). The quantitative expressions for TLS briefly summarized here are taken from ref (5).

#### Sound Pressure, Wavelength, and *L*

Sound pressure oscillates sinusoidally in one dimension with amplitude  and phase shift ϕ.
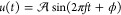
1The frequency of oscillator *f* in Hz is converted to radial frequency ω (radians s^–1^) as ω = 2*πf*. The ratio of the speed of sound in the fluid *c* and the frequency determine the wavelength λ of the sound pressure wave (cm).
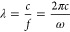
2

The optimal cell thickness *L* to establish constructive interference is defined relative to the wavelength.^4,5^
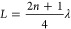
3There are infinitely many *n* solutions for a given λ, where the electrode is best placed at the extremes (maxima and minima) of the sinusoidal pressure wave. However, as *n* increases and *L* increases, the wave attenuates and energy is diminished. For ultrasonic oscillators of 10 to 60 kHz, λ and so *L* are on the order of a centimeter.

#### Solvent Properties

The properties of the solvents determine important acoustic phenomena. Speed of sound *c* (cm/s) is a property of the solvent. The ratio of solvent viscosity η and density ρ set the kinematic viscosity ν. A List of Symbols is provided. Calculated parameters for the specific solvents are listed in Table 1.
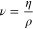
4Acoustic impedance *Z* is a mass flux set by the density and speed of sound in a material. *Z* characterizes the fluids and solids.
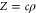
5

**Table 1:** Solvent Properties, Transmittance and Attenuation Parameters, and Relative Energies^a^

	THF	DMF	water	EtOH	2-propanol
**Solvent Properties**					
density ρ (g/cm^3^)	0.889	0.944	0.997	0.816	0.785
viscosity η (cp)	0.55	0.92	0.8921	1.1	2.86
kinematic viscosity ν (cm^2^/s)	0.00619	0.00975	0.00895	0.0135	0.0364
dielectric constant ϵ (20 °C)	7.6	36.7	78.54	24.55	18.3
surface tension γ (mN m^–1^)	26.4	36.8	72.8	22.3	21.8
compressibility (Pa)	10.1	6.4	4.6	11.2	10.0
p*K*_auto_	34.70^24^	28	13.997	18.8	20.8
molar volume *V*_M_ (cm^3^ mol^–1^)	81.1	73.3	19.1	56.5	76.6
speed sound (m/s)	1289	1457.5	1498	1144	1139^25^
speed of sound *c* (cm/s)	128,900	145,750	149,800	114,400	113,900
acoustic impedance *Z* (g/cm^2^ s)	114,592	149,351	137,588	93,350	89,412
reflectance *R*_solvent | Pt_	0.96	0.95	0.95	0.97	0.97
**Attenuation Parameters**					
spatial α (cm^–1^); as *αf*^–2^ (10^–18^ s^2^ cm^–1^)	1.93	2.10	1.77	6.00	16.44
temporal *k* (rad s^–1^); as *kf*^–2^ (10^–12^ rad s)	1.56	1.92	1.67	4.31	11.76
**Transmittance Parameters**					
*Tf*^2^ (10^15^ s^–2^)	1.91	1.55	1.79	0.692	0.254
*T* at *f* = 41 kHz (10^6^)	1.14	0.92	1.06	0.41	0.15
**Relative Energies**					
*V*_M_*T*_solvent_/*V*_M_*T*_water_	4.55	3.34	1.00	1.15	0.57

a Unless otherwise noted, values are from references for density, viscosity, dielectric constant,^26,27^ speed of sound,^26,28^ and autoprotolysis constant.^29^

#### Reflectivity at the Interface

Reflectivity describes the fraction of the energy reflected from the interface relative to the energy incoming from the interface. Because sound travels more efficiently in denser media such as solid versus liquid, reflection of the sound energy back into the fluid is necessary to build an interfacial constructive interference. Reflectivity *R*_1|2_ is characterized by the acoustic impedance of electrode material and the solvent.
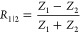
6Here, the electrodes are platinum, where *c*_Pt_ = 268,000 cm s^–1^ and ρ_Pt_ = 21.47 g cm^–3^. *Z*_Pt_ = *c*_Pt_ ρ_Pt_ = 5,754,000 g cm^–2^ s^–1^. Then,
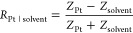
7Because *Z*_Pt_ is large compared to *Z*_solvent_, *R*_Pt | solvent_ approaches 1.

#### Sound Attenuation

As sound pressure propagates through the fluid, the pressure is attenuated with distance and time. For a given solvent, spatial attenuation α (cm) is specified by solvent properties ν and *c* and the oscillator frequency.
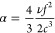
8

The temporal attenuation *k* (cm^2^ s^–1^) is proportional to the spatial attenuation and the speed of sound.
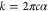
9

On solution of the partial differential equations for the propagation of *u*(*x, t*),^5^ the roots of the equation are set by a coefficient defined as β_*n*_, where . The natural frequencies of the system for *n* = 0, 1, 2, 3, ... are ω_*n*_ = (2*n* + 1)*πc*(2*L*)^−1^. Temporal attenuation *k* and natural frequency of the system ω_*n*_ set β_*n*_. Resonance and constructive interference are established where the system is underdamped. That is, where β_*n*_ > 0, ω_*n*_/2 exceeds temporal attenuation.

#### Transmissibility and Amplification

Where the system is underdamped and natural frequencies suffice to overcome temporal attenuation, transmissibility *T* is established. Transmissibility is amplification. The product of transmissibility and temporal attenuation for water is a constant that extends to all solvents. See Figure 7 in ref (5).
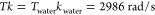
10Transmissibility is dimensionless and on the order of 10^6^ in a well-constructed thin layer cell.

#### Energy

The energy input to the interface varies with the solvent. The input energy is proportional to the interfacial pressure drop and the molar volume, *V*_M_ of the solvent. In water, the energy delivered per second (the power) for a 25 kHz oscillator is ≈1 kJ (mol s)^−1^ across the cross section of the oscillator and the electrode. Irradiating the interface for a minute delivers ≈60 kJ mol^–1^ of energy in water. Experimentally, this suffices to remove the oxide layer on platinum in about 15 min.^3^ The energy delivered for a given solvent is proportional to the product of the molar volume and transmissibility. The energy for a given solvent relative to water at any frequency is *V*_M_*T*_solvent_*f*^2^ normalized by *V*_M_*T*_water_*f*^2^. The relative energies are reported in Table 1 as *V*_M_*T*_solvent_/*V*_M_*T*_water_. In a well-built thin layer cell, sound pressure energy in THF and DMF are 4.6 and 3.3 that of water; EtOH is comparable (1.15 ×) to water; and 2-propanol is 60% the energy of water.

### Solvent Properties

The characteristic equations rely on the bulk properties of the solvents, as defined in the List of Symbols and Abbreviations. The bulk properties of the five solvents and several parameters relevant to characterizing the propagation of sound in a fluid are summarized in Table 1. General solvent properties of dielectric constant ϵ, surface tension γ, compressibility, autoprotolysis constant *pK*_auto_, and molar volume *V*_M_ are also listed.

Transmittance *T* is the amplification of sound pressure. *T* is dimensionless and of the order of 10^6^. Values in Table 1 are for a well-designed cell; optimal conditions are diminished by cell misalignment.^2^

## Experimental Section

The experimental setup is presented in ref (23) for nonaqueous solvents, a modification of the cell design presented previously for water.^2,3^ The thin layer cell is shown in Figure 1. The meniscus forms proud of the top of the hollow cylinder as a hemisphere to an oblate hemisphere, as determined by the solvent surface tension γ. In these experiments, all cells are formed with the same *L*/λ. The cells were not configured to maximize the transmissibility. The quasireference electrode is a platinum wire inside a glass capillary. The entire cell is housed in a larger container, where the atmosphere is saturated solvent vapor in nitrogen.

### Solvents and Redox Probes

Nonaqueous electrochemical solvents used in this study are tetrahydrofuran (THF, (CH_2_)_4_O), dimethylformamide (DMF, (CH_3_)_2_NCH), ethanol (EtOH, CH_3_CH_2_OH), 2-propanol (CH_3_CHOHCH_3_) (Fisher Scientific), and water. The redox probes selected for electroactivity and solubility in the nonaqueous solvents were iron(III) nitrate nonahydrate (Fe(NO_3_)_3_·9H_2_O, 99.99%, Sigma-Aldrich) and p-benzoquinone (BQ, ≥98%, Sigma-Aldrich). Redox probes were 1.00 mM in nonaqueous solvents with 0.10 M tetrabutylammonium tetrafluoroborate (TBABF_4_, Sigma-Aldrich). In water, the electrolyte is 0.10 M H_2_SO_4_. Tris(bipyridine)ruthenium(II) dichloride (Ru(bpy)_3_Cl_2_, Fisher Scientific) was evaluated in water with a sulfuric acid electrolyte. Electrolytes were degassed with nitrogen presaturated with solvent vapor for 30 min prior to measurements and held under a nitrogen blanket during measurements. The thin layer cell is held inside a container that contains solvent-saturated vapor.

### Configured TL Cell

In the thin layer sonoelectrochemical cell,^2,3^ an ultrasonic transducer (400ET18S, Pro-Wave Electronic Corporation) fitted into a Teflon shroud served as the bottom of the cell. The function generator (4011A, 5 MHz, BK Precision) is matched to the 41 kHz frequency of the oscillator, and voltage is applied to drive maximum oscillator intensity. A small volume (∼1 mL) of degassed electrolyte containing redox probe is pipetted into the hollow cylinder cell to form a meniscus proud of the top face of the Teflon cylinder, as shown in Figure 1. The meniscus forms as a hemisphere to an oblate hemisphere dependent on the surface tension γ. Three platinum wires (0.5 mm diameter, 99.95%, Alfa Aesar) are inserted into the fluid parallel to the face of the transducer but not touching the transducer. Wires are positioned at about 120^o^ separation and extend into the fluid approximately 2.0 cm for an electrode area of about 0.3 cm^2^. The three wires serve as the working, counter, and quasi-reference electrodes that are connected to a CH Instruments 760B potentiostat. The quasireference is oxide on platinum, where the oxide can be removed on sonication to shift the potential of the QRE. To mitigate against oxide removal, the quasireference is placed inside a glass capillary. Care is taken to electrically isolate the potentiostat and function generator, which are on separate electrical circuits. The cell is placed inside a secondary container that is purged with solvent vapor-saturated nitrogen throughout the measurements. In water, no change in temperature (Δ*T* ≲ 1 °C) is measured and no cavitation is observed by the eye. On visual inspection in nonaqueous solvents, neither cavitation nor evaporation is observed for any solvent.

### Voltammetry

Cyclic voltammograms are recorded at 50 mV s^–1^ before sonication (PreSono), during sonication (Sono) at 0, 1, 5, and 10 min, and after sonication ceases (PostSono) at 0, 1, 5, and 10 min. Ferric ion and BQ are reduced over voltage ranges shown in the CVs. Ru(bpy)_3_^2+^ is oxidized. All electron transfers are taken as single electron transfers. Because the stability of the quasireference potential is uncertain, peak potentials are reported against the quasireference, but potentials are not thermodynamically significant. Measurements of peak or maximum current, differences in the peak potentials Δ*E*_peak_, and the rise of current on the forward sweep toward the peak (Δ*i*/Δ*E*) are used to characterize the kinetics. All measurements are single replicates. The hemispherical configuration is used for each of the solvents. No attempt to optimize the cell thickness relative to the wavelength of the oscillator was made for the individual solvents.^2^ The model to be tested is in 1 dimension for parallel plates, but the thin layer cell configuration is hemispherical. In the hemisphere, the sound pressure is thought to reflect back from the meniscus at the electrolyte | air boundary to establish constructive interference at the electrode | electrolyte interface. In the thin layer cell, the meniscus stands about 1 cm above the transducer, and the electrodes are placed at half height in the hollow cylinder. Correlations of transmittance (amplification) in the solvents are anticipated to have analogous dependence on solvent parameters despite the different electrode geometries.

## Results

To evaluate the impacts of the solvent on TLS, cyclic voltammograms are evaluated for five solvents at 50 mV s^–1^ at Pt wire electrodes with 41 kHz quartz crystal oscillators. The solvent sits as a sessile volume above the oscillator with three Pt wire electrodes in the electrolyte, parallel to and above the plane of the oscillator (Figure 1). No cavitation is observed. Two probes with less than facile interfacial electron transfer rates are evaluated: Fe^3+^ and BQ. Peak and maximum currents are measured. Mechanistic changes are noted by visual inspection of CV morphologies. A Pt wire serves as a quasi-reference electrode. Because sonication removes oxides from the platinum quasireference, potential differences are well-defined but potential values at peak currents are not reliable.

For the four nonaqueous solvents, currents increase on sonication (Sono) and after sonication ceases (PostSono) as compared to data collected before the onset of sonication (PreSono). PostSono currents are generally higher than Sono currents. The sole exception for the nonaqueous solvents is BQ in DMF where electron transfer is fast, and an increase in interfacial sound pressure does not further increase the electron transfer rate. CVs in water differ in some ways from nonaqueous solvents. Results are presented separately for Fe^3+^ and BQ. Notations such as Sono10 and PostSono5 denote 10 min after the onset of sonication and 5 min after sonication ceased. Voltammetric currents correlate with the transmissibility of the solvents listed in Table 1. Throughout, the model predicts *T*, consistent with the experimental results, to vet the model for nonaqueous solvents.

### Fe(NO)_3_

The reduction of ferric ions is a one electron process.
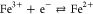
11Because the number of waters of hydration for ferric and ferrous ions differ substantially, the electron transfer rate for the electrolysis in aqueous solution is slowed as waters of hydration are lost and gained. The standard heterogeneous electron transfer rate *k*^0^ is characterized as quasi-reversible  to irreversible . Unlike Ru(bpy)_3_^2+^, where the electron transfer is fast compared to mass transport , in quasireversible and irreversible heterogeneous electron transfer, the rate of electron transfer is comparable to or lower than the mass transfer rate.

**Figure 3:**
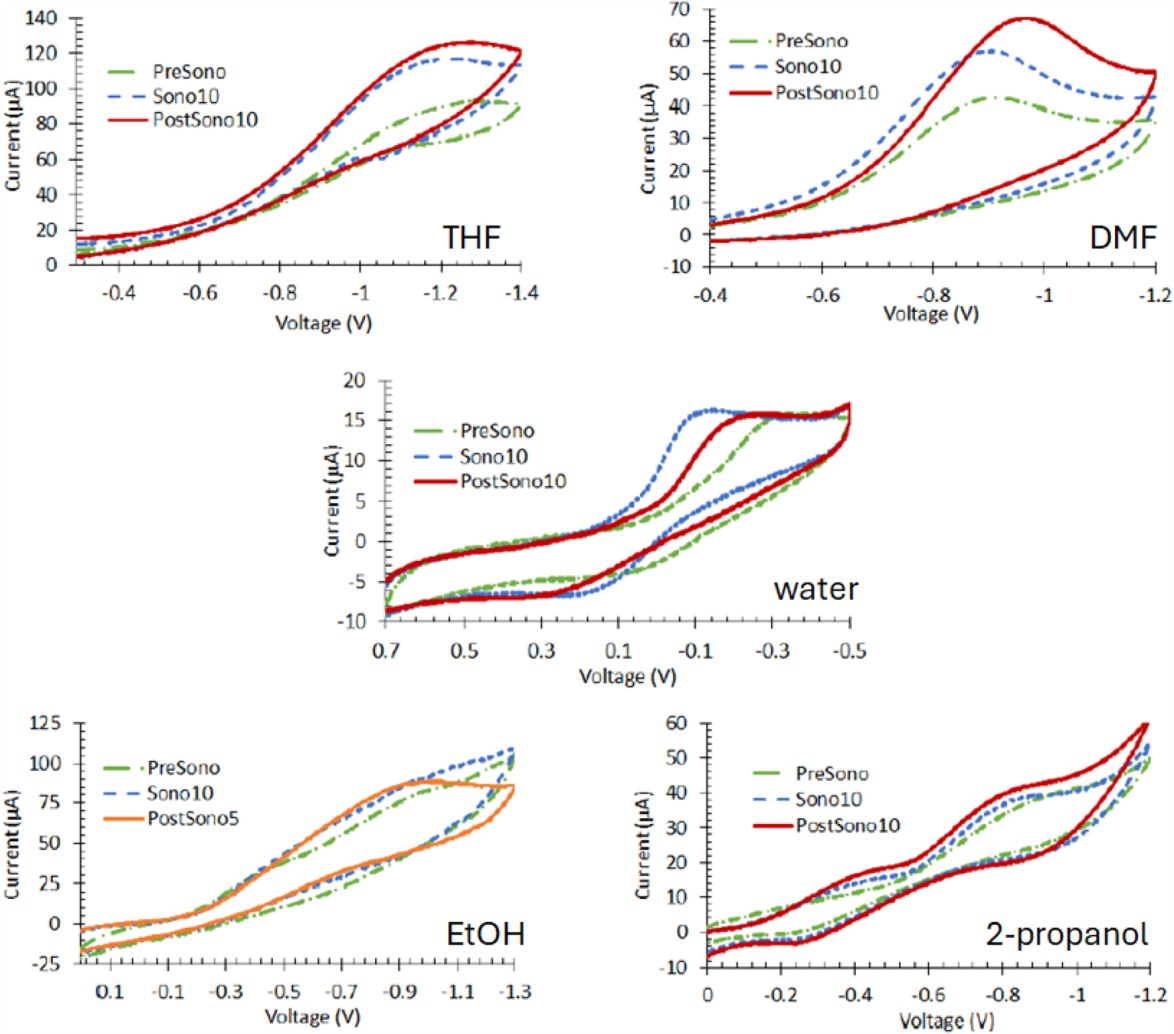
Cyclic voltammograms recorded at 50 mV s^–1^ at Pt wire electrodes are shown for 1.00 mM Fe(NO_3_)_3_ in the four nonaqueous solvent that contain 0.10 M TBABF_4_ electrolyte. In water, the electrolyte is 0.10 M H_2_SO_4_. In all solvents, Sono10 (blue dashed line) and PostSono (solid lines) currents and electron transfer rates are higher than under PreSono (green dot dash) conditions. In all cases, sound pressure impacts persist for at least 10 min after sonication ceases. For the nonaqueous solvents, PostSono currents are higher than those of Sono10 with comparable electron transfer rates. For water, the currents PostSono10 are comparable to Sono10, but the electron transfer rate is slower based on the rise of Δ*i*/Δ*E*.

In Figure 3, cyclic voltammograms recorded at Pt wire electrodes at 50 mV s^–1^ for 1.00 mM Fe(NO_3_)_3_ in THF, DMF, EtOH, and 2-propanol with 1.0 M TBABF_4_ and aqueous 0.10 M H_2_SO_4_ are shown. On visual inspection before sonication (PreSono, green ·−), the reactions are best characterized as  in all solvents. After 10 min of sonication (Sono10, blue dash), forward peak currents are increased; the rise toward the peak potential (Δ*i*/Δ*E*) is steeper for all but EtOH. An increase in peak current and steepness of Δ*i*/Δ*E* mark increase in the heterogeneous electron transfer rate.

However, with the possible exception of water, all electron transfer rates remain characterized as  based on peak splitting (Δ*E*_peak_ ≳ 200 mV). Return waves are only observed in water and 2-propanol; in THF, DMF, and EtOH, the oxidation of Fe^2+^ back to Fe^3+^ is likely positive of the starting potential because the electron transfer is extremely slow. For the alcohols, small evidence of an additional electron transfer is noted at −0.4 V, but the discussion here focuses on the larger forward peak current of −0.8 V.

The impact of sonication persists for at least 10 min after sonication ceases. Post-sonication voltammograms (PostSono, at 10 min solid red, at 5 min solid orange) have higher currents and faster electron transfer rates than the PreSono samples. In THF, DMF, EtOH, and 2-propanol, PostSono compared to Sono10 peak currents are higher and Δ*i*/Δ*E* is comparable to Sono10. In water, peak currents are comparable, but the electron transfer rate is lower for PostSono10 as compared to Sono10.

Peak currents are summarized in Table 2 for PreSono, Sono at 0, 1, 5, and 10 min, and PostSono at 0, 1, 5, and 10 min. In all solvents except water, peak currents increase with sonication and post-sonication. The maximum currents are recorded at the last PostSono sample. The fraction of maximum enhancement is found as the maximum current normalized by the PreSono maximum current for a given solvent. For the nonaqueous solvents, enhancements are ordered THF ≳ DMF > EtOH > 2-propanol. No replicate measurements were made for the nonaqueous solvents with either Fe^3+^ or BQ. However, across all TLS measurements^1−3,23^ peak current consistently scales as PreSono < Sono ≲ PostSono. There is statistical consistency across the nonaqueous solvents for Fe^3+^ and BQ as the results are shown to be linearly consistent with the TLS model. There is no evidence of a change in temperature. In SI.3, the equivalent temperature *T*_eqv_ needed to increase the peak current by a given fraction is estimated. From the Randles Sevcik equation for  cyclic voltammetric peak current, as *i*_p_ increases, *T*_eqv_ decreases.^30,31^ Currents are enhanced by factors of 1.1 in 2-propanol to 1.8 in THF that correspond to *T*_eqv_ values of −47 and −201 °C. For typical enhancements for all five solvents, *T*_eqv_ is below the freezing point of the solvent. The enhanced peak currents on sonication are not due to a change in temperature (Table SI.1).

**Table 2:** Peak or Maximum Currents (μA) for 1.00 mM Fe(NO_3_)_3_ in Five Solvents Are Shown for Data before Sonication (PreSono), during Sonication (Sono) for 0, 1, 5, and 10 min from Onset of Sonication, and Post-Sonication (PostSono) for 0, 1, 5, and 10 min after Sonication Ceased^a^

time (min)		THF	DMF	Water	EtOH	2-Prop
	PreSono	37.0	22.6	7.2	24.0	7.2
0	Sono	45.5	24.7	6.3	24.2	8.0
1	Sono1	47.8	25.4	**8.8**	23.6	8.0
5	Sono5	53.7	29.9	7.0	23.7	8.0
9.9	Sono10	54.6	33.8	6.7	24.1	7.4
10.1	PostSono	56.7	30.5	6.7	23.3	8.2
11	PostSono1	63.2	34.9	6.9	30.1	8.1
16	PostSono5	65.5	38.5	6.6	**30.9**	8.1
21	PostSono10	**67.5**	**40.2**	5.6		**8.2**
	Frac. Max. Enhance.	**1.82**	**1.78**	**1.22**	**1.29**	**1.13**

a Maximum currents are boldfaced for each solvent. With the exception of water, the final PostSono current is highest. The maximum fraction of current enhancement is the highest current normalized by the current PreSono. Enhancements are largest in THF and DMF with the highest relative energies in Table 1.

**Figure 4:**
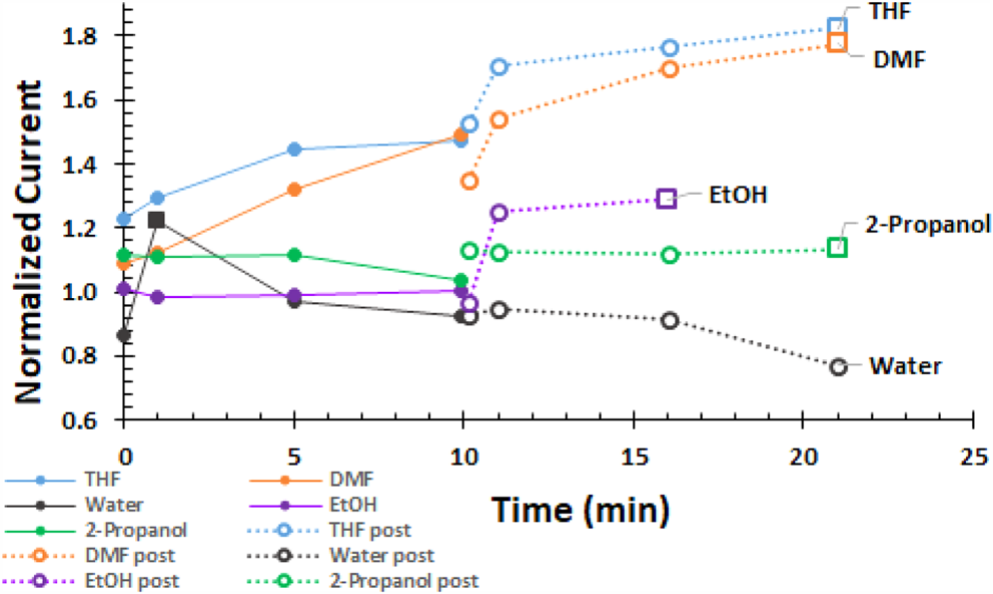
Current normalized by the PreSono peak current for Fe^3+^ is shown as a function of time as the system is sonicated (Sono) for 0, 1, 5, and 10 min (filled circles) and then for 0, 1, 5, and 10 min after sonication ceases (PostSono). Except for water, currents are higher for Sono and PostSono. Impact of sonication persists PostSono. In all but water, normalized current is highest for the last PostSono sample. The largest effects are in THF and DMF.

The voltammograms are recorded in an uninterrupted sequence at times listed in Table 2. The time evolution of the peak or maximum currents is shown in Figure 4. The currents are normalized by the PreSono peak current for each solvent. For the nonaqueous solvents, the normalized current at Sono0 is at least 1. With the exception of water, the normalized current increases with time. PostSono currents (open circles) are higher than Sono currents. The largest enhancements are for THF and DMF, with smaller enhancements observed for the alcohols, as anticipated by the model.

From the TLS Model, solvent properties impact current on sonication. The enhancement increases as η, ν, and attenuations α and *k* decrease. Enhancement tends to be larger with higher ρ, *c*, and p*K*_auto_. There is no apparent correlation with ϵ, γ, and *V*_M_, or compressibility. The relative energy, the sound pressure energy, in the solvent relative to water is *V*_M_*T*_solvent_/*V*_M_*T*_water_. For Fe^3+^, the normalized current scales linearly with the relative energy.

**Figure 5:**
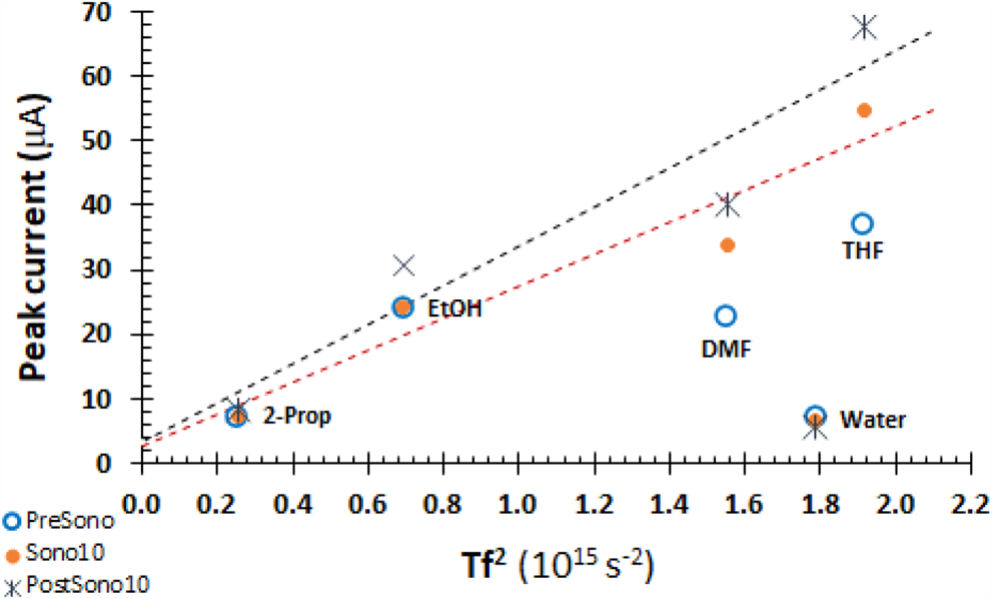
Peak current for Fe^3+^ correlates with solvent property *Tf*^2^ or equivalently with transmissibility *T* for the 41 kHz oscillator. PreSono peak currents (empty circles) are uncorrelated with *Tf*^2^. Excluding water, correlations for Sono10 (orange circles) and PostSono (*) are apparent. For THF, DMF, EtOH, and 2-propanol, regression of peak current (μA) with *Tf*^2^ (10^15^ s^–2^) yields *y* = (24.7 ± 5.2)*x* + (2.7 ± 6.7) with *R*^2^ = 0.92 for Sono10, or slope of 25 × 10^–21^ A s^2^ and intercept of 0. For PostSono10, *y* = (30.3 ± 7.5)*x* + (3.2 ± 9.6) with *R*^2^ = 0.89, or slope of 30 × 10^–21^ A s^2^ and intercept of 0. PostSono currents are perhaps 20% higher than Sono10. The peak currents for PostSono10 are higher than for Sono10, consistent with the build of energy introduced by sonication after sonication ceases.

From eq 10, the transmissibility (amplification) *T* scales with *k*^–1^, where *k* depends on *c*, ν = η/ρ, and *f* in eqs 8 and 9. *Tf*^2^ is characteristic of each solvent and oscillator frequency. All measurements are made with a 41 kHz oscillator. Values of *Tf*^2^ and *T* are shown in Table 1. In Figure 5, peak currents are plotted against *Tf*^2^. PreSono currents (open circles) are not correlated with *Tf*^2^, but as η^–1/2^ as expected in the quiescent electrolyte (SI.4). Impacts of sonication on peak current in water are negligible, as shown in Table 2 and Figure 3. For the Sono10 data (orange circles), there is a good correlation of peak current with *Tf*^2^. Similarly, for PostSono data (*), the peak current correlates with *Tf*^2^. The energy introduced as sound pressure by ultrasound and constructive interference at the electrode | electrolyte interface persists for at least 10 min (PostSono10). The slope for PostSono10 is higher than for Sono10, with marginal statistical significant. The intercepts are zero.

### BQ

The reduction of BQ is a two electron process with two coupled protons possible. The reactant Q represents BQ and hydroquinone H_2_Q is the fully protonated, reduced product.
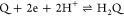
12

The square scheme presents the nine possible species for the two proton two electron reduction.^32−34^ Sequential reductions are left to right; sequential protonations are top to bottom. Availability of protons impacts the path for BQ reduction. Water with sulfuric acid provides the largest proton concentration. In the nonaqueous solvents, inherent availability of proton is highest in the alcohols followed by DMF and THF, tracked as the autoprotolysis constants (Table 1). Depending on the available protons, quinone voltammograms can exhibit one or two peaks with corresponding 2 or 1 electrons transferred. Here, the number of electrons transferred is taken as 1. The nine species square scheme represents numerous proton-dependent paths from quinone to hydroquinone, which complicates comparisons between the solvents.

**Scheme 1:**
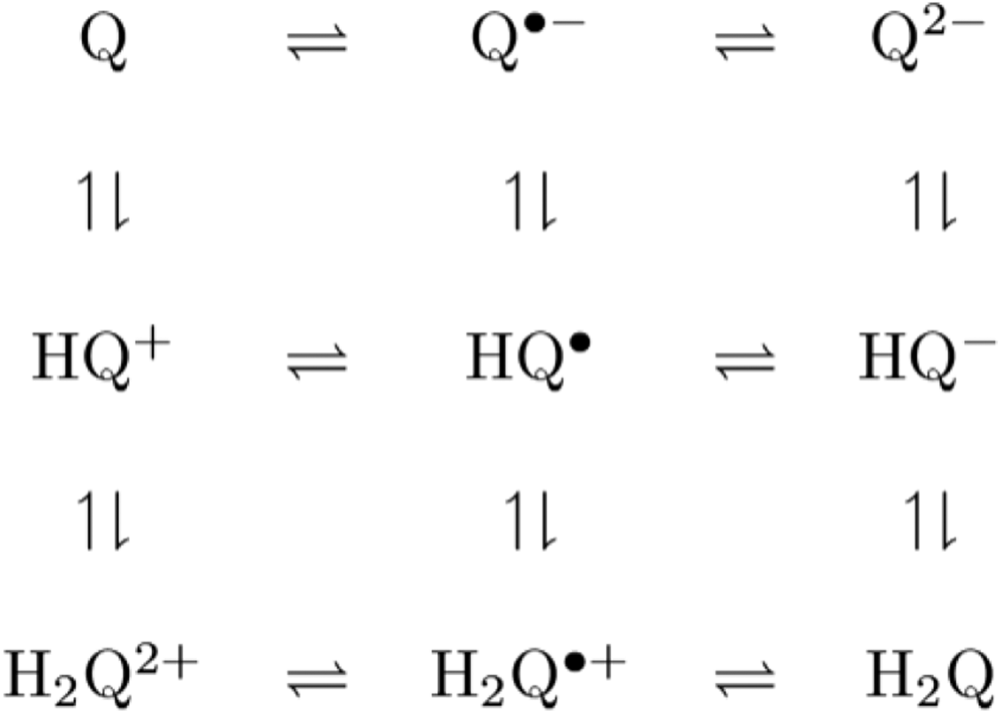
Square Scheme for Benzoquinone (Q) Reduction and Protonation

**Figure 6:**
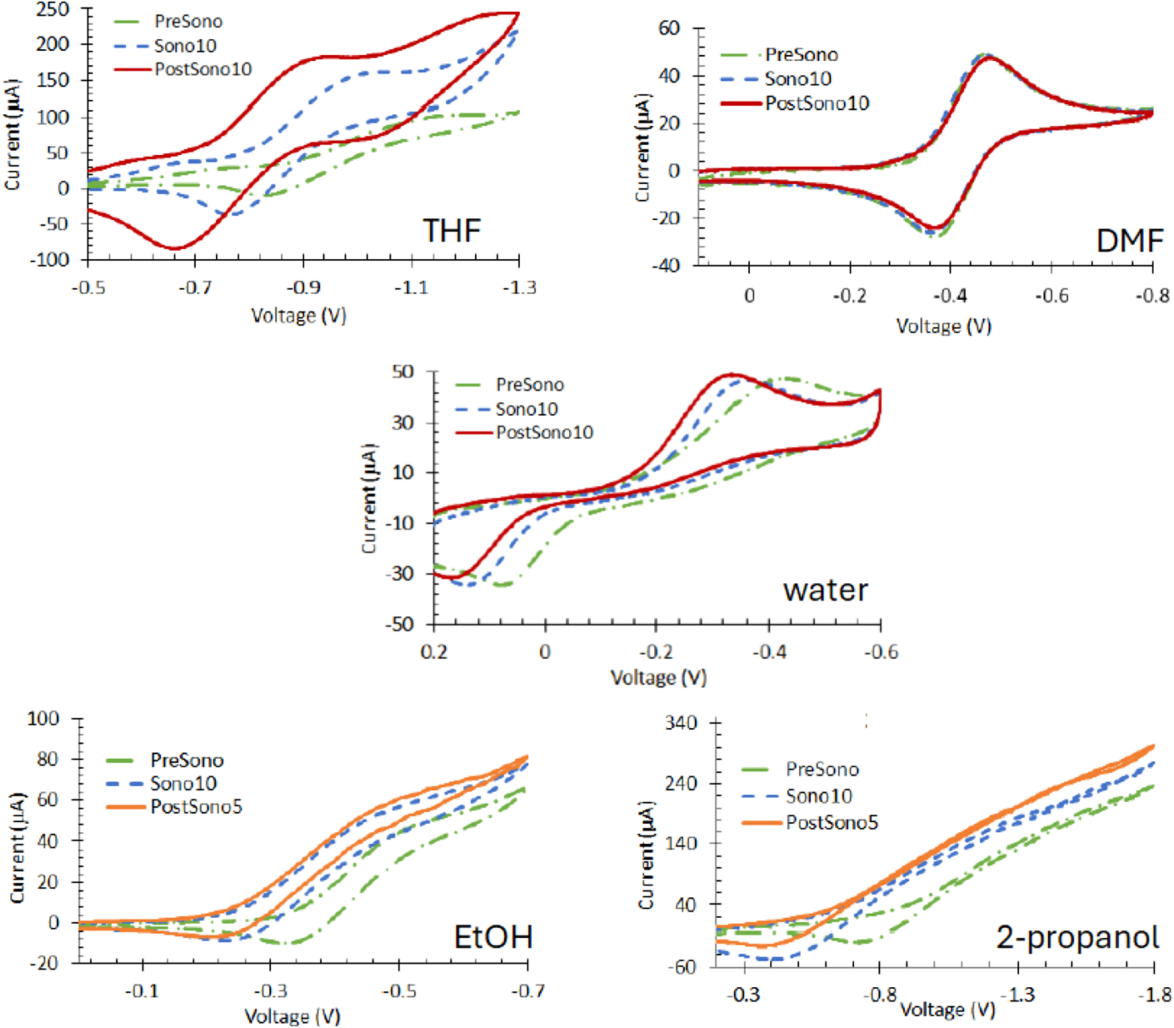
Cyclic voltammograms recorded at 50 mV s^–1^ at Pt wire electrodes are shown for 1.00 mM BQ in the four nonaqueous solvent that contain 0.10 M TBABF_4_ electrolyte. In water, the electrolyte is 0.10 M H_2_SO_4_. Mechanisms vary. In THF and water, electron transfer is slow . In DMF, electron transfer is fast . For the alcohols, a coupled, facile protonation step may affect the voltammetric morphology. In all solvents except DMF, currents and electron transfer rate increase with sonication as compared to PreSono (green dot dash). For THF and water, impacts of PostSono (solid lines) are slightly higher than those of Sono (dashed lines). For alcohols, impacts are less substantial. In these four solvents, effects persist for at least 10 min after sonication ceases. Kinetics in DMF are fast PreSono, so the rates are not increased by sonication.

Cyclic voltammograms recorded at 50 mV s^–1^ for 1.00 mM BQ at Pt wire electrodes are shown in Figure 6. On visual inspection, the voltammograms have different mechanistic characteristics. In THF, the electron transfer reaction shifts from  to approach  on sonication. In DMF, BQ undergoes rapid electron transfer  that is unaffected by sonication. In water, the electron transfer is slow  with an increase in the rate on sonication; there is some asymmetry to the voltammogram, perhaps consistent with a transfer coefficient other than 0.5. In the alcohols, the voltammograms are distinctly shaped, and the mechanism is less clear. In EtOH, the sigmoidal voltammograms may indicate a facile, coupled preceding chemical reaction such as protonation. In 2-propanol, the voltammograms are morphologically more similar to those of EtOH but less well differentiated. Mechanisms for BQ are known to be dependent on the availability of proton.^32−34^ For the nonaqueous solvents, the proton concentration is highest in the alcohols and lowest in THF, as estimated from p*K*_auto_ constants. The sigmoidal CV morphology for EtOH and 2-propanol is consistent with a  mechanism, a chemical step  preceding the electron transfer step , where , a rate-determining step, is a proton transfer. Autoprotolysis of EtOH and 2-propanol limits the concentration of the available protons.

In THF, the electron transfer rate increases with sonication and a substantial increase in peak current. The peak current is larger for PostSono10 than Sono10. From Δ*i*/Δ*E* of the current rise, the electron transfer rate is higher with sonication, where the post-sonication electron transfer rate is marginally faster. In aprotic THF and DMF, reduction proceeds from *Q* to *Q*^•–^ to *Q*^2–^. In DMF, the electron transfer rate and protonation reactions are fast. As in Ru(bpy)_3_^2+^ in Figure SI.2, electron transfers fast compared to mass transport  are not made faster on sonication. In water, the peak current increases on sonication with PostSono10 currents slightly higher than Sono10 currents. From Δ*i*/Δ*E*, the electron transfer rates are comparable to Sono and PostSono. Sonication impact on the alcohols is less, where sonication increases the maximum current slightly and Δ*i*/Δ*E* very slightly. The overall smaller effect of sonication on alcohols may arise from a coupled preceding protonation reaction.

Peak and maximum currents for BQ are tabulated in Table 3 for THF, DMF, water, and EtOH. With no readily identifiable peak currents, 2-propanol is not shown. DMF is little impacted by sonication because electron transfer is fast compared to mass transport. Maximum current increases on sonication of BQ in THF, water, and EtOH, with THF the largest impacts and water and EtOH comparable, THF ≫ water ∼ EtOH. In these three solvents, post-sonication currents are higher than the final sonication current. The fraction of maximum enhancement is ordered as THF ≫ water ∼ EtOH, with the largest enhancements found at PostSono10. In DMF, the current is not enhanced. As for Fe^3+^, no replicate measurements were made for BQ. The observed effects of sonication on THF are substantial, on EtOH and water are small, and on 2-propanol approach negligible. These results are consistent with those of the TLS model.

**Table 3:** Peak Currents (μA) for 1.00 mM BQ in Four Solvents Are Shown PreSono, at 0, 1, 5, and 10 min Sono, and PostSono^a^

time (min)		THF	DMF	Water	EtOH
	PreSono	27.4	46.3	27.8	22.1
0	Sono	36.7	46.0	26.4	23.7
1	Sono1	32.6	45.0	26.6	22.8
5	Sono5	48.8	42.7	29.5	24.1
9.9	Sono10	49.4	42.1	31.8	24.8
10.1	PostSono	51.7	**44.5**	27.8	26.3
11	PostSono1	51.5	44.4	28.5	26.5
16	PostSono5	58.4	41.8	33.0	24.9
21	PostSono10	**59.5**	40.3	**33.9**	**27.8**
	Frac. Max. Enhance.	**2.17**	**0.96**	**1.22**	**1.26**

a Maximum currents and maximum fractional enhancement are boldfaced. Enhancements rank as THF ≫ water ∼ EtOH, with little impact in DMF.

**Figure 7:**
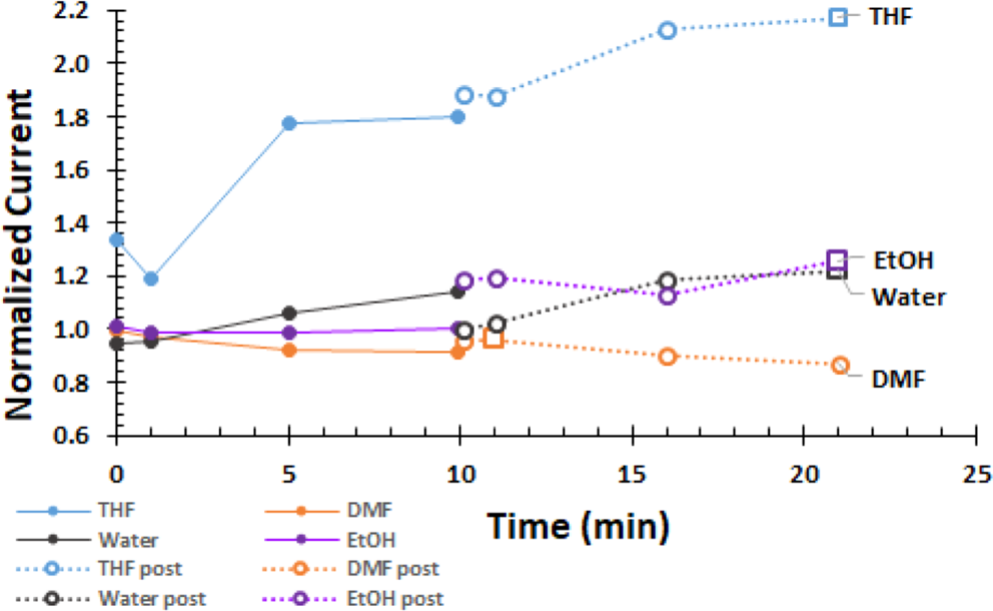
Normalized current for 1.00 mM BQ is shown with time for sequential sonication (Sono, filled circles) followed immediately by post-sonication (PostSono, open circles), each at 0, 1, 5, and 10 min. For THF, EtOH, and water, currents increase with sonication, with higher post-sonication currents. Impacts in THF are largest. DMF data are little affected by sonication because the electron transfer rate for BQ in DMF is fast and not augmented further by sonication.

The normalized current with time and sonication is shown in Figure 7. Excluding DMF, sonication increases current with post-sonication currents higher than sonication currents. Impacts are the largest in THF.

For THF, water, and EtOH, enhancement increases as η, ν, and *k* decrease. For only three solvents, enhancement does not correlate with α, ρ, *c*, p*K*_auto_, ϵ, γ, or compressibility. For BQ in THF, water, and EtOH, the normalized current scales linearly with the relative energy.

**Figure 8:**
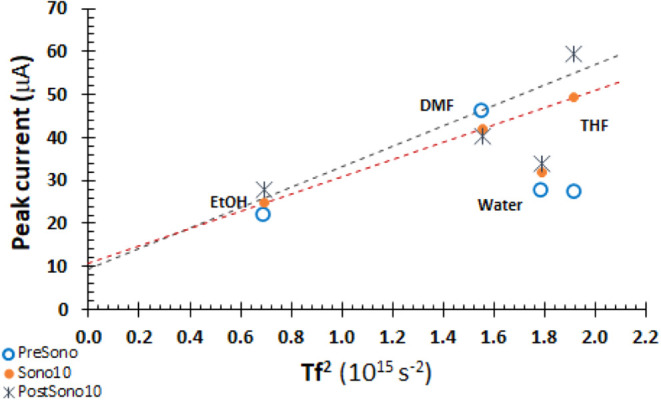
BQ Peak current for PreSono data (open circles) are uncorrelated with *Tf*^2^ or equivalently with transmissibility *T* for the 41 kHz oscillator. For THF, DMF, and EtOH, peak and maximum currents correlate with *Tf*^2^ for Sono10 and PostSono10 data. For Sono (orange), regression of peak current (μA) with *Tf*^2^ yields *y* = (21.12 ± 0.00)*x* + (10.87 ± 0.00) with *R*^2^ = 1.00 for Sono10, or slope of 21.12 × 10^–21^ A s^2^ and intercept of 10.87 × 10^–6^ A. For PostSono (blue), the regression is weaker, *y* = (23.8 ± 8.8)*x* + (9.4 ± 13.1) with *R*^2^ = 0.88, or slope of 24 × 10^–21^ A s^2^ and intercept of 0. Regressions excluded water data.

In Figure 8, the peak or maximum current is plotted against *Tf*^2^. No correlation for PreSono data is apparent, consistent with the varied reaction paths of Scheme 1. Sonication of EtOH, DMF, and THF electrolytes increases with *Tf*^2^ for Sono and PostSono data. Linearity for the Sono data is good. Water with 0.1 M sulfuric acid is off line. In all solvents, energy is introduced to the electrode | electrolyte interface as sound pressure that persists for at least 10 min post-sonication.

## Discussion

From the TLS model, constructive interference is achieved where cell thickness *L* and oscillator wavelength λ are matched according to eq 3. There are multiple solutions for *L* ∝ λ with *L* = λ/4 as the first. Constructive interference focuses sound pressure energy at the electrode | electrolyte interface to increase slow interfacial rates.^4−6^ From the data, interfacial rates are increased for Fe^3+^ and BQ in all nonaqueous solvents, with the exception of BQ in DMF where electron transfer is fast. In all studies, no cavitation is observed, consistent with prior experiments in water.^1−3^ A major objective of this work is to vet the TLS model in solvents other than water.

### Fast Electron Transfer

Consistent with prior experiments,^2^ sonication did not impact voltammograms (Figure SI.2) for Ru(bpy)_3_^2+^ in water. In an outer sphere redox probe, electron transfer for Ru(bpy)_3_^2+^ in water is fast, , compared to the mass transport rate. For fast interfacial rates compared to mass transport, sonication does not further increase interfacial rates. In Figure 6 for BQ in DMF, the voltammogram is unchanged by sonication because the interfacial reaction rate is fast. From the voltammetry for BQ in DMF and Ru(bpy)_3_^2+^ in water, sonication does not impact mass transport. The match of *L* and λ suffices to deposit energy at the electrode | electrolyte interface but does not impart fluid motion (convection) in the thin layer. Because the solvent is quiescent under thin layer conditions, quantitative voltammetric measurements allow assessment of reaction pathways and rates that are not possible in classical sonoelectrochemistry.

### Mechanisms

For all samples except BQ in DMF, sonication improves interfacial rates for Fe^3+^ (Figure 3) and BQ (Figure 6) as marked by increased peak or maximum currents, decreased Δ*E*_peak_, and on the advance of the forward sweep, steeper rise of Δ*i*/Δ*E*. Because sonication can remove oxide from the Pt | Pt oxide quasireference electrode, peak potential and shifts in peak potentials are not reliable markers of kinetics. For Fe^2+^, mechanisms are slow electron transfer mechanisms,  to perhaps  for Sono10 in water. For BQ, different mechanistic pathways are accessible. As shown in the Square Scheme, availability of protons and electrons alter reaction rates and mechanisms. The apparent mechanism in THF and water is ; in DMF, fast as ; and in the alcohols, the mechanism may be a preceding chemical step of protonation . For BQ, sonication impacts are larger in THF with no protons and water with the most protons and smaller in the alcohols, where proton concentration limits the interfacial rate.

### PostSono Observation

In all cases except BQ in DMF and Fe^3+^ in water, current recorded after sonication ceased (PostSono) is higher than the highest currents recorded on sonication at Sono10, shown in Tables 2 and 3. Plots of maximum or peak currents normalized by the PreSono maximum currents with approximate time track the enhancement with the sonication history for Fe^3+^ in Figure 4 and BQ in Figure 7. Normalized current grows with time where last PostSono is higher than Sono10. Largest enhancements are in THF. In light of the model and the experimental data, it is noted sound pressure energy input to the electrode | electrolyte interface persists post-sonication; the input sound pressure energy builds after sonication ceases; and the current enhancement persists for at least 10 min post-sonication. In water, PostSono persists approaching 30 min is observed.^3^

From the model, during sonication, energy increases at the electrode | electrolyte interface when sound pressure is well reflected.^5^ That the rate enhancements persist and increase PostSono suggests that a kinetic event lags the input of sound pressure energy at the interface during Sono. That PostSono persists for at least as long at the sonication suggests the energy is input to the electrode rather than the more fluxional electrolyte. At Pt in water, the energy input to the interface is estimated as ∼1 kJ (mol s)^−1^ based on the model.^4,5^ Experimentally from CV, Pt oxide layers are removed from the electrode in ∼20 min.^3^ Oxides are at least partially removed under similar conditions for W and Al electrodes.^3^ For Ag electrodes, sonication intensity is sufficient to free Ag^+^ into the electrolyte, where the concentration increases with time sonicated.^3^ Carbon pencil electrodes disintegrate on sonication.^3^ For different electrodes, sonication changes the composition and perhaps the crystal face of the electrode surface. The energy input to the surface integrates with time; at ∼1 kJ (mol s)^−1^ integrated over 20 min, can deliver an ∼1 MJ of energy mol^–1^ to the interface if there are no losses. This is sufficient energy to remove oxides and restructure crystal faces. If the processes to reform the electrode surface lag energy input during sound pressure oscillations, then the integrated energy input may be effectively accumulated PostSono to change the electrode surface and increase currents.

### PostSono Verification

In Tables 2 and 3, the maximum fractional enhancement is the highest current normalized by the PreSono current for each solvent. The enhancements in each solvent are comparable for Fe^3+^ and BQ. Largest enhancements are in THF. The model anticipates an increase in rate (peak or maximum faradaic current) with *T*, transmissibility (amplification). From the model, *T* is found from eq 10 and temporal attenuation is found in eq 9. In Table 1, *T* is shown for the 41 kHz oscillator used in the experiments, and for all frequencies as *Tf*^2^. Experimental peak currents are plotted against model defined *Tf*^2^ for Fe^3+^ in Figure 5 and BQ in Figure 8. For both probes, the patterns are the same. PreSono is not correlated with *Tf*^2^. Sonication increases current, and current persists post-sonication. For nonaqueous solvents, current under sonication and post-sonication increases linearly with *Tf*^2^. With limited statistical significance, the calculated slopes are 15 to 20% higher for the postsonicated samples. Thus, the model is vetted by experimental data for nonaqueous solvents, consistent with the buildup of sound pressure energy at the electrode | electrolyte interface.

### Linear Correlation with Model

**Figure 9:**
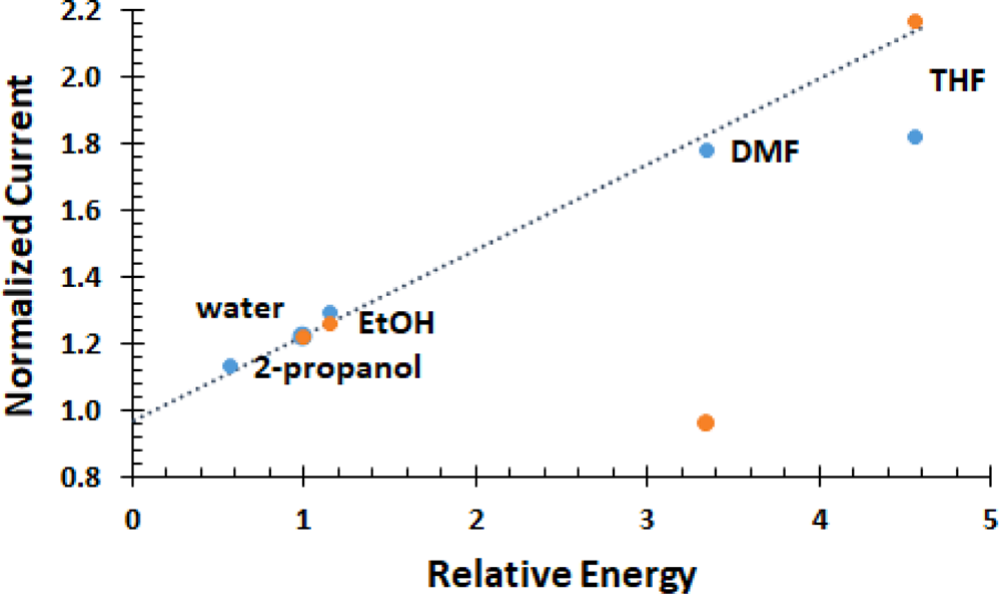
Experimental normalized current (maximum fractional enhancement) is plotted against the energy inherent to solvents calculated from the model. The relative energy is normalized by the energy inherent to water, *V*_M_*T*_solvent_/*V*_M_*T*_water_. With the exception of BQ in DMF  and Fe^3+^ in THF, all data are linearly correlated. For Fe^3+^ (blue) except THF, *y* = (0.234 ± 0.009)*x* + (1.00 ± 0.02) with *R*^2^ = 0.997 for four points. For BQ except for DMF  (orange), *y* = (0.2672 ± 0.0003)*x* + (0.953 ± 0.0008) with *R*^2^ = 0.99999 for three points. The regression line for seven points (black dots) is y = (0.256 + 0.008) x + (0.97 + 0.02) with *R*^2^ = 0.995.

An underlying concept of thin layer sonochemistry is that energy deposited at the electrode | electrolyte interface varies with solvent properties. The energy inherently available for a given solvent relative to water *V*_M_*T*_solvent_/*V*_M_*T*_water_ is calculated from literature solvent properties and tabulated in Table 1. The relative energies are several-fold higher for THF and DMF compared to water where the alcohols are comparable to water. Normalized current is the maximum enhancement for a given probe in a given solvent. In Figure 9, the experimental normalized current is plotted against relative energy for the solvent, as calculated from the model. With the exception of BQ in DMF and Fe^3+^ in THF, regression for seven points yields *y* = (0.256 ± 0.008)*x* + (0.97 ± 0.02) with *R*^2^ = 0.995. The maximum fractional enhancement is proportional to one fourth of the relative energy. The enhancement is linearly and strongly correlated with the properties of the solvent defined as the relative energy, *V*_M_*T*_solvent_/*V*_M_*T*_water_. Thus, consistent with the TLS model, the rate enhancement correlates linearly with the sound pressure energy that can be built at the electrode | electrolyte interface by sonication to induce constructive interference in underdamped systems.

### Sensitivity to Experimental Setup

The model is built for parallel faces of the electrode and sonicator, where sound pressure reflects off the electrode | electrolyte interface. The experiments are in a hemispherical geometry, where the sound pressure reflects off the electrolyte | air interface. Despite the differences in geometry, the experimental properties are well predicted by the solvent parameters in the model. Transmissibility in TLS is sensitive to alignment and the relationship between *L* and λ, which is solvent-dependent.^4^ Here, *L*/λ is not optimized, but current is enhanced. There is no visible cavitation and enhancements rank as PreSono < Sono ≲ PostSono, as recorded in aqueous systems. Although the thin layer cell was not optimized for each solvent, enhancements were tracked with relative energy. Attention to cell design may yield greater enhancements. It is noted that constructive interference is established for cells with less than optimal configurations and that the enhanced rates still track with solvent properties as anticipated by the model.

### Applications in Sonochemistry

Finally, TLS applications are not limited to electrochemistry. Where constructive interference can be established in a thin fluid layer, thin layer sonochemistry and increased interfacial rates can be established. This spans a range of frequencies. Ultrasound sonochemistry is used to make nanomaterials. In a matrix without frank cavitation, better control of the nanomaterial sizes may be possible. Sea salt aerosol (SSA) forms from ocean spray to introduce particulate to the atmosphere,^35^ where the particulate can serve as heterogeneous catalysts.^36^ Absent cavitation, constructive interference in a fluid layer may contribute to SSA formation at longer wavelengths. Sonochemistry is used in electrodeposition, where thin layer conditions that eliminate cavitation may lead to improved plate quality at higher deposition rates.

## Summary

Thin layer sonoelectrochemistry is undertaken with nonaqueous solvents (THF, DMF, EtOH, and 2-propanol) and redox probes Fe^3+^ and BQ.^23^ Constructive interference is established on sonication to enhance the rates of slow interfacial reactions. No cavitation and no heating of the thin fluid layer is noted. The solutions are quiescent, which allows for quantitative voltammetric measurements under sonochemical conditions. It is noted that currents are enhanced on sonication as compared to before sonication. Effects of sonication persist for at least 10 min after the sonicator is extinguished. For nonaqueous solvents, currents for postsonicated samples are higher than those for sonicated currents.

A previously developed model for TLS quantifies amplification as transmittance *T*.^5^*T* is specified based on the solvent properties. Experimental peak or maximum currents scale linearly with *T* at a single frequency. Maximum fractional enhancements are similar for Fe^3+^ and BQ in a given solvent. The model is vetted quantitatively against the voltammetric data for the four nonaqueous solvents. The model specifies the inherent energy available on sonication for each solvent as energy relative to water. Rate enhancements (current normalized by presonicated current) for the two redox probes depend linearly on the relative energies of the solvents, *V*_M_*T*_solvent_/*V*_M_*T*_water_. The largest relative energy is found for THF where the largest enhancements are measured.

Nonaqueous solvents can be used to focus energy at the electrode | electrolyte interface to increase rates for otherwise slow interfacial reactions. Where constructive interference is established, the model and solvent properties allow for the prediction of effective conditions for interfacial rate enhancement. TLS provides a physiochemical means to accelerate interfacial rates. Major advantages of thin layer sonoelectrochemistry and sonochemistry are (1) fundamental studies of sonicated fluids with no cavitation and (2) applications where focused interfacial sound pressure impacts rates at substantially lower energy input and no heating of the bulk fluid.

## Supporting Information Available

Simplified diagram of the hemispherical TLS cell; cyclic voltammograms of the outer sphere  for Ru(bpy)_3_^2+^ in water under TLS conditions; estimate of the change in temperature for equivalent current enhancements; and relationship between peak current and viscosity in quiescent PreSono electrolytes (PDF)

## Symbols and Abbreviations

ρ: density of solvent or electrode material (g cm^–3^)
η: viscosity of solvent g (s cm)^−1^ (cp)
ν: kinematic viscosity of solvent ν = η/ρ (cm^2^ s^–1^)
*V*_*M*_: molar volume of solvent; *V*_M_ = *MW*/ρ (cm^3^ mol^–3^)
*c*: speed of sound in the solvent (cm s^–1^)
*Z*: acoustic impedance of material *Z* = *cρ*; mass flux (g cm^–3^ s^–1^)
*R*_1|2_: reflectivity at the solvent|electrode interface; *R*_1|2_ = (*Z*_1_ – *Z*_2_)/(*Z*_1_ + *Z*_2_)
ϵ: dielectric constant of the solvent at 20 °C
γ: surface tension of the solvent (mN m^–1^)
p*K*_auto_: –log *K*_auto_ where *K*_auto_ is solvent autoprotolysis constant
*f*: oscillator frequency in Hz (s^–1^)
ω: radial oscillator frequency in radians per second; ω = 2*πf* (radian s^–1^)
*T*: sound pressure transmittance (amplification)
α: spatial attenuation of sound pressure in solvent (cm)
*k*: temporal attenuation of sound pressure in solvent (cm^2^ s^–1^)
*L*: thickness of thin fluid layer (cm)
λ: wavelength of ultrasound; λ = *c*/*f* = 2*πc*/ω (cm)
*u*(*x,t*): sound pressure at position *x* at time *t* (Pa)
amplitude of the sound pressure input by the oscillator (Pa)
ϕ: phase shift in the sound pressure wave
*k*^0^: standard heterogeneous electron transfer rate (cm s^–1^)
