# Supplementary material for: Thin Layer Sonoelectrochemistry: The Solvents
Source: J Phys Chem C Nanomater Interfaces. 2025 Feb 28;129(10):5197–208. doi: 10.1021/acs.jpcc.4c08175 (PMC11912532; doi:10.1021/acs.jpcc.4c08175)
Supplement: Supplementary file 1 — jp4c08175_si_001.pdf [file jp4c08175_si_001.pdf]

Supplemental Information for Thin Layer Sonoelectrochemistry: The Solvents

Nadeesha P.W. Rathuwadu, Daniel L. Parr IV, and Johna Leddy*

University of Iowa
Department of Chemistry
Iowa City, IA 52240 USA
johna-leddy@uiowa.edu

Abstract

In thin layer sonoelectrochemistry (TLS), ultrasound induces constructive interference in a thin fluid layer to increase interfacial rates. In TLS experiments, slow interfacial rates are increased during and after sonication. No cavitation and no heating are observed in the fluid. A previously developed model quantifies how solvent properties impact TLS rates. Voltammetry for Fe^{3+} and benzoquinone in tetrahydrofuran, dimethylformamide, water, ethanol, and 2-propanol is undertaken with and without sonication. Rate enhancements vary with solvent properties as quantitatively predicted by the model. The data vet the TLS model in nonaqueous solvents.

Table of Contents for SI

SI.1: Simplified Diagram of the Hemispherical TLS Cell used in the experiments SI.2
SI.2: Cyclic Voltammograms for $Ru(bpy)_3Cl_2$ in water under TLS conditions SI.3
SI.3: Estimate of Temperature Equivalence for Peak Current Enhancement SI.4
SI.4: Quiescent Peak Current Increase and Viscosity η SI.5

SI.1 Simplified Diagram of the Hemispherical TLS Cell

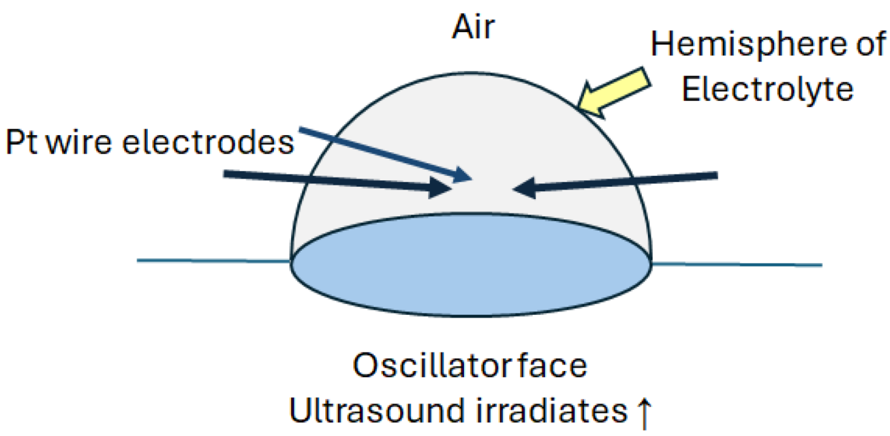

Figure SI.1: The hemispherical TLS used in the cyclic voltammetric measurements is formed by pipetting the electrolyte solution over the oscillator of about 2 cm diameter. The QCO is embedded into a polymer shroud. A roughly hemispherical meniscus of electrolyte sits atop the face of the QCO. Three Pt wires inserted at about half height and parallel to the face of the QCO serve as working, counter, and quasireference electrodes (QRE). The QRE is inserted in a narrow capillary to try to minimize loss of Pt oxides during sonication. The electrolyte is degassed with nitrogen prior to measurement. The cell is placed inside a container, the atmosphere of which is degassed over the course of the measurement.

SI.2 Cyclic Voltammograms for $Ru(bpy)_3Cl_2$ in water under TLS conditions

On thin layer sonication of $Ru(bpy)_3Cl_2$ in aqueous electrolyte, changes to the voltammetric response are minimal, as observed previously. [2,3] $Ru(bpy)_3Cl_2$ undergoes rapid, outer sphere interfacial electron transfer. Energy input as sound pressure does not increase the interfacial electron transfer rate. There is also no impact of thin layer sonication on mass transport.

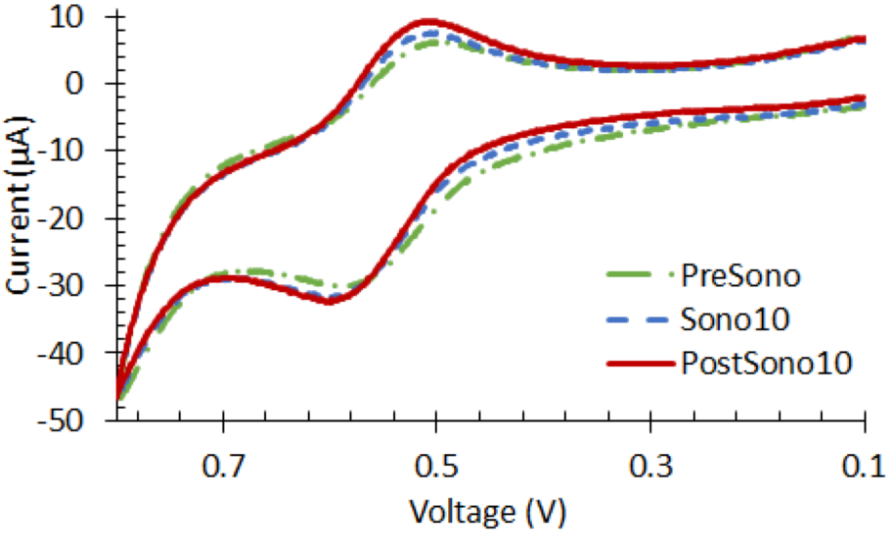

Figure SI.2: Cyclic voltammograms recorded at Pt wire electrodes at 50 mV/s for 1.00 mM $Ru(bpy)_3^{2+}$ in 0.100 M aqueous H_2SO_4 before sonication (PreSono, green dash-dot), during sonication after total sonication time was 10 min (Sono10, blue dash), and at 10 min after sonication was stopped (PostSono10, red solid). The voltammograms are coincident about the faradaic process, as consistent with no impact of constructive interference on the rates of either electron transfer or mass transport. The fluid is quiescent.

SI.3 Estimate of Temperature Equivalence for Peak Current Enhancement

Consider the peak current i_p for a fast electron transfer, $\mathbb{E}_{rev}$. The Randles Sevcik equation applies, eqn (6.2.18) in *Electrochemical Methods*. [30] This equation is developed by Nicholson and Shain. [31] For quasireversible and irreversible electron transfers, the equations differ, but the functional dependence on temperature is similar.

$$i_p = 0.4463(nF)^{3/2}R^{-1/2}AD^{1/2}c^*v^{1/2}T^{-1/2} \quad \text{(SI.1)}$$

All parameters constant except T, i_p *decreases* as T increases. Current enhancement ratios R were found in the range of 1.1 to 1.8. The equivalent temperature T_{eqv} for a given R is:

$$T_{eqv}(K) = \frac{298}{R^2} \quad \text{(SI.2)}$$

For $R = 1.1$, T_{eqv} is 246 K; for $R = 1.4$, T_{eqv} is 152 K; for $R = 1.8$, T_{eqv} is 92 K. For the enhancements found for the five solvents, the equivalent temperature to achieve R enhancements is below the freezing point of the solvent in many conditions.

	THF	DMF	Water	EtOH	2-Prop
Common Frac. Max. Enhance. (R)	**1.8**	**1.8**	**1.2**	**1.3**	**1.1**
T_{eqv} (K)	92.0	92.0	206.9	176.3	246.3
T_{eqv} (C)	-201	-201	-86	-117	-47
T_{freeze} (C)	-108.4	-61	0.0	-114.6	-89

Table SI.1: Equivalent temperature T_{eqv} to drive the commonly observed fractional maximum enhancements in the five solvents. The freezing point T_{freeze} (C) of the solvents is shown. All the solvent freezes before T_{eqv} is reached for common values of R.

SI.4 Quiescent Peak Current Increase and Viscosity η

In quiescent solutions, i_p scales with $D^{1/2}$, where D is the diffusion coefficient, as in equation SI.1 for $\mathbb{E}_{rev}$. Viscosity η and D are inversely proportional, such that i_p is linear with $\eta^{-1/2}$.

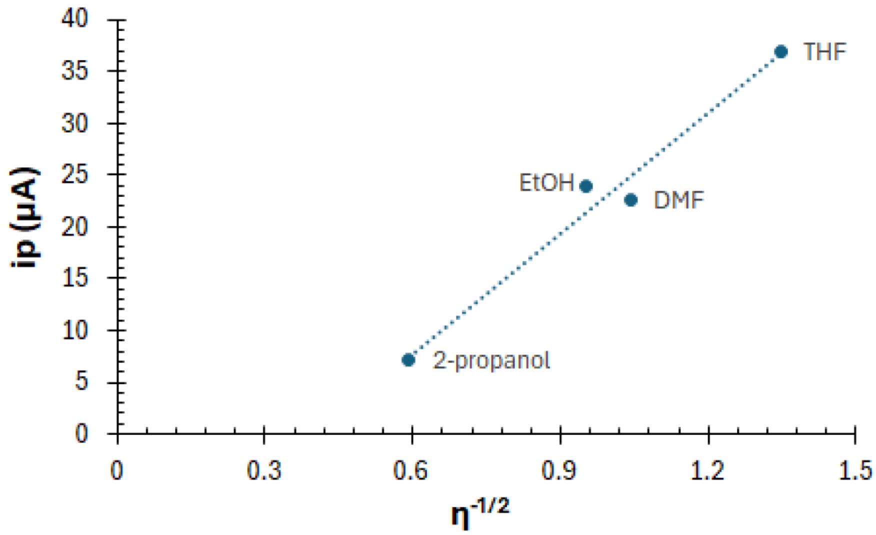

Figure SI.3: Peak currents i_p in quiescent (PreSono) solutions correlate with $D^{1/2}$ and so $\eta^{-1/2}$ where η is viscosity. Here, for Fe^{3+} in the nonaqueous solvents is linear as $i_p(\mu A) = (38.6 \pm 4.5)\eta^{-1/2} - (15.3 \pm 4.6)$ with $R^2 = 0.97$.

References

[1] J. Leddy, C. G. Duda, J. Lyon, and I. Leddy, William J., "Thin layer sonochemistry and sonoelectrochemistry devices and methods," *US Patent 10,300,453*, 2019. Assignee: J. Leddy.

[2] C. G. Duda, B. Parke, and J. Leddy, "Thin layer sonoelectrochemistry: Impact on slow heterogeneous electron transfer," *Electrochimica Acta*, vol. 468, p. 143118, 2023.

[3] C. G. Duda, *Thin Layer Sonoelectrochemistry.* Ph.D., University of Iowa, 2012, 2012.

[4] D. L. Parr IV and J. Leddy, "To configure thin layer sonoelectrochemical experiments," *Journal of The Electrochemical Society*, vol. 168, no. 6, p. 066524, 2021.

[5] D. L. Parr IV, C. G. Duda, and J. Leddy, "Why Sonochemistry in a Thin Layer? Constructive Interference," *The Journal of Physical Chemistry C*, vol. 127, pp. 12184–12193, jun 16 2023. [Online; accessed 2023-07-22].

[6] D. L. Parr IV, *Optimization in Electrochemistry: New Methods in Sonoelectrochemistry, Electrochemical Separations, and Education.* Ph.D., University of Iowa, 2022.

[7] J. P. Lorimer and T. J. Mason, "Sonochemistry: Part 1 - the physical aspects," *Chem. Soc. Rev.*, vol. 16, pp. 239–274, 1987.

[8] A. G. Wallace and M. D. Symes, "Decoupling strategies in electrochemical water splitting and beyond," *Joule*, vol. 2, no. 8, pp. 1390–1395, 2018.

[9] J. Riesse, T. Caulier, C. Deckerheer, O. Fabre, J. Vandercammen, J. L. Delplancke, and R. Winand, "Quantitative sonochemistry," *Ultrasonics Sonochemistry*, vol. 3, pp. S147–S151, 1996.

[10] R. J. Wood, J. Lee, and M. J. Bussemaker, "A parametric review of sonochemistry: Control and augmentation of sonochemical activity in aqueous solutions," *Ultrasonics sonochemistry*, vol. 38, pp. 351–370, 2017.

[11] F. Touyeras, J. Hihn, X. Bourgoin, B. Jacques, L. Hallez, and V. Branger, "Effects of ultrasonic irradiation on the properties of coatings obtained by electroless plating and electro plating," *Ultrasonics sonochemistry*, vol. 12, no. 1-2, pp. 13–19, 2005.

[12] W. L. Ang, P. J. McHugh, and M. D. Symes, "Sonoelectrochemical processes for the degradation of persistent organic pollutants," *Chemical Engineering Journal*, vol. 444, p. 136573, 2022.

[13] J. Theerthagiri, J. Madhavan, S. J. Lee, M. Y. Choi, M. Ashokkumar, and B. G. Pollet, "Sonoelectrochemistry for energy and environmental applications," *Ultrasonics Sonochemistry*, vol. 63, p. 104960, 2020.

[14] B. Pollet, J.-Y. Hihn, M.-L. Doche, J. Lorimer, A. Mandroyan, and T. Mason, "Transport limited currents close to an ultrasonic horn: equivalent flow velocity determination," *Journal of the Electrochemical Society*, vol. 154, no. 10, p. E131, 2007.

[15] S. J. Coleman and S. Roy, "Design of an ultrasonic tank reactor for copper deposition at electrodes separated by a narrow gap," *Ultrasonics Sonochemistry*, vol. 42, p. 445–451, Apr. 2018.

[16] K. Yasui, "Numerical simulations for sonochemistry," *Ultrasonics Sonochemistry*, vol. 78, p. 105728, 2021.

[17] R. G. Compton, J. C. Eklund, and F. Marken, "Sonoelectrochemical processes: a review," *Electroanalysis*, vol. 9, no. 7, pp. 509–522, 1997.

[18] Y. T. Didenko, W. B. McNamara, and K. S. Suslick, "Temperature of multibubble sonoluminescence in water," *The Journal of Physical Chemistry A*, vol. 103, no. 50, pp. 10783–10788, 1999.

[19] K. S. Suslick, Y. Didenko, M. M. Fang, T. Hyeon, K. J. Kolbeck, W. B. McNamara III, M. M. Mdleleni, and M. Wong, "Acoustic cavitation and its chemical consequences," *Philosophical Transactions of the Royal Society of London. Series A: Mathematical, Physical and Engineering Sciences*, vol. 357, no. 1751, pp. 335–353, 1999.

[20] K. S. Suslick, D. A. Hammerton, and R. E. Cline, "Sonochemical hot spot," *Journal of the American Chemical Society*, vol. 108, no. 18, pp. 5641–5642, 1986.

[21] E. B. Flint and K. S. Suslick, "The temperature of cavitation," *Science*, vol. 253, no. 5026, pp. 1397–1399, 1991.

[22] S. J. Doktycz and K. S. Suslick, "Interparticle collisions driven by ultrasound," *Science*, vol. 247, pp. 1067–1068, 1990.

[23] N. P. W. Rathuwadu, *Magnetic Field Effects on Electrochemical Systems, Lanthanide Electrochemistry, Thin Layer Sonoelectrochemistry, and Models for Polymer Film Characterization.* PhD thesis, University of Iowa, 2017, 2017.

[24] J. Barbosa, D. Barrón, and S. Butí, "Autoprotolysis Constants and Standardization of pH Measurements in Tetrahydrofuran-Water Mixtures," *Electroanalysis*, vol. 11, pp. 627–631, 7 1999. [Online; accessed 2024-11-12].

[25] A. B. Pereiro and A. Rodríguez, "Thermodynamic Properties of Ionic Liquids in Organic Solvents from (293.15 to 303.15) K," *Journal of Chemical & Engineering Data*, vol. 52, pp. 600–608, jan 25 2007. [Online; accessed 2024-11-13].

[26] T. M. Aminabhavi and B. Gopalakrishna, "Density, Viscosity, Refractive Index, and Speed of Sound in Aqueous Mixtures of N,N-Dimethylformamide, Dimethyl Sulfoxide, N,N-Dimethylacetamide, Acetonitrile, Ethylene Glycol, Diethylene Glycol, 1,4-Dioxane, Tetrahydrofuran, 2-Methoxyethanol, and 2-Ethoxyethanol at 298.15 K," *Journal of Chemical & Engineering Data*, vol. 40, pp. 856–861, 7 1995. [Online; accessed 2024-11-07].

[27] "Physical Properties of Solvents." https://www.sigmaaldrich.com/deepweb/assets/sigmaaldrich/marketing/global/documents/614/456/labbasics_pg144.pdf?srsltid=AfmBOoooWIlPI8_L1g0volblkX17ixGZ8lRak_KIRxxZzqku1yyQZ6eI. [Online; accessed 2024-11-12].

[28] E. Edge, "Speed of Sound in Various Liquids and Solids." https://www.engineersedge.com/physics/speed_of_sound_16034.htm. [Online; accessed 2024-11-12].

[29] S. Rondinini, P. Longhi, P. R. Mussini, and T. Mussini, "Autoprotolysis constants in nonaqueous solvents and aqueous organic solvent mixtures," *Pure and Applied Chemistry*, vol. 59, pp. 1693–1702, jan 1 1987. [Online; accessed 2024-11-12].

[30] A. Bard and L. Faulkner, *Electrochemical Methods.* New York: John Wiley & Sons, Inc., second ed., 2001.

[31] R. Nicholson and I. Shain, "Single scan and cyclic methods applied to reversible, irreversible, and kinetic systems," *Anal. Chem.*, vol. 36, pp. 706–723, 1964.

[32] E. Laviron, "Electrochemical reactions with protonations at equilibrium: Part viii. the 2 e, 2h+ reaction (nine-member square scheme) for a surface or for a heterogeneous reaction in the absence of disproportionation and dimerization reactions," *Journal of Electroanalytical Chemistry and Interfacial Electrochemistry*, vol. 146, no. 1, pp. 15–36, 1983.

[33] E. Laviron, "Electrochemical reactions with protonations at equilibrium: Part x. the kinetics of the p-benzoquinone/hydroquinone couple on a platinum electrode," *Journal of Electroanalytical Chemistry and Interfacial Electrochemistry*, vol. 164, no. 2, pp. 213–227, 1984.

[34] E. Laviron, “Electrochemical reactions with protonations at equilibrium: Part xii. the 2 e-, 2 h+ homogeneous isotopic electron exchange reaction (nine-member square scheme),” *Journal of Electroanalytical Chemistry and Interfacial Electrochemistry*, vol. 169, no. 1-2, pp. 29–46, 1984.

[35] R. E. Cochran, O. S. Ryder, V. H. Grassian, and K. A. Prather, “Sea spray aerosol: The chemical link between the oceans, atmosphere, and climate,” *Accounts of Chemical Research*, vol. 50, p. 599–604, Mar. 2017.

[36] T. H. Bertram, R. E. Cochran, V. H. Grassian, and E. A. Stone, “Sea spray aerosol chemical composition: elemental and molecular mimics for laboratory studies of heterogeneous and multiphase reactions,” *Chemical Society Reviews*, vol. 47, no. 7, p. 2374–2400, 2018.
